# A Remarkable Case of Uterine Hæmorrhage

**Published:** 1781-08

**Authors:** 


II. *A remarkable cafe of uterine hæmorrhage. By* Thomas Blackburne, M. D. F. R. S. *phyfician at Durham. Read* July 16, 1781.

M. B. a married woman aged 46, of a thin, fpare habit, by trade a cook, had for fome

ſome years ſcorbutic eruptions on her arms, hands, and fingers, which, being detrimental to her in her profeſſion, ſhe had repelled with ſome ſtrong ointment ſeveral times. From the begining of the year 1778 theſe eruptions returned no more, but ſhe was troubled with frequent and large floodings for about a year.

An exceſſive diſcharge of this kind happened to her about the middle of July 1779, attended with a ſenſe of bearing down *ex anteriori*, and very ſevere acute pains in her left hypogaſtrium, which continued with very little intermiſſion for three weeks, attended with looſeneſs and vomiting. She deſcribed them as being not like labour-pains, but more ſevere, and ſaid that they always began on the left ſide. She was treated as for an inflammation of the bowels with bleeding, &c. About the third week ſhe found that ſhe had paſſed by the vagina a ſmall, round ſubſtance about the ſize of a plum, which ſhe compared to a little bag full of ſomething tied up, and at the ſame time an offenſive putrid maſs hanging from the vagina. In this ſituation ſhe continued ſome days, with a very copious putrid diſcharge daily from the maſs, and was then removed 20 miles in a poſt-chaiſe to Durham, where I firſt ſaw her on the 1ſt of September 1779. I found her greatly reduced by the disease, and the room, in which she was, so intolerably offensive that it was scarcely possible to stay in it, though it was full of flowers and the window wide open. The discharge was considerably abated, and there was a membranous substance hanging out six or eight inches from the vagina, the end of which was dry and like a piece of the dried coat of a bladder; nearer the vagina moist and discharging putrid matter. I ordered her a strong decoction of the bark to be taken frequently, and visited her the next day with an eminent surgeon and man-midwife of this city. He examined her, and found the projecting body extend up the vagina as far as his hand could reach, but growing thicker and thicker the higher it went. He could not satisfy himself of its extent or attachment, but proposed a ligature to be made as high in the vagina as possible upon this substance to bring it away.

The next day, however, it had come away spontaneously, and proved to be a large, putrid, indistinct mass, weighing about three pounds; of a round shape, (like the fundus uteri) at its base, of about 4½ inches in its largest diameter, and tapering almost regularly into the membrane above described, with which it was covered throughout.

The

The covering membrane was thin, and the fubftance through it appeared convoluted like the lobes of the cerebrum mixed with coagulated blood; there appeared no inner cavity, but the fubftance was in too diffolved and putrid ftate to be defcribed particularly.

The woman recovered her health every day from this delivery, and has been very regular and enjoyed her health fince that time much better than before. She never complained of any extraordinary fullnefs during the difeafe, nor of any difference in her fize and feelings fince.

Cafes in fome refpects fimilar to this are to be met with in books; but the coming on of the flooding after the repulfion of a cutaneous eruption, and the extraordinary bulk of the coagulum (for fuch it feems to have been) are circumftances perhaps not undeferving the attention of the Society. Ruyfch has obferved, that blood coagulated in the uterus has often been miftaken for a falfe conception, and that by compreffion it has frequently acquired a firm, and even membranous appearance. Obfervations of the fame nature occur likewife in Morgagni and other practical writers.

*Durham*, July 1, 1781.

SECTION

